# Frataxin overexpression in Müller cells protects retinal ganglion cells in a mouse model of ischemia/reperfusion injury *in vivo*

**DOI:** 10.1038/s41598-018-22887-5

**Published:** 2018-03-19

**Authors:** Rowena Schultz, Melanie Krug, Michel Precht, Stefanie G. Wohl, Otto W. Witte, Christian Schmeer

**Affiliations:** 1 Department of Ophthalmology, Jena University Hospital, Jena, Germany; 2 Hans-Berger Department of Neurology, Jena University Hospital, Jena, Germany; 3 Department of Biological Structure, University of Washington Seattle, Seattle, United States

## Abstract

Müller cells are critical for retinal function and neuronal survival but can become detrimental in response to retinal ischemia and increased oxidative stress. Elevated oxidative stress increases expression of the mitochondrial enzyme frataxin in the retina, and its overexpression is neuroprotective after ischemia. Whether frataxin expression in Müller cells might improve their function and protect neurons after ischemia is unknown. The aim of this study was to evaluate the effect of frataxin overexpression in Müller cells on neuronal survival after retinal ischemia/reperfusion in the mouse *in vivo*. Retinal ischemia/reperfusion was induced in mice overexpressing frataxin in Müller cells by transient elevation of intraocular pressure. Retinal ganglion cells survival was determined 14 days after lesion. Expression of frataxin, antioxidant enzymes, growth factors and inflammation markers was determined with qRT-PCR, Western blotting and immunohistochemistry 24 hours after lesion. Following lesion, there was a 65% increase in the number of surviving RGCs in frataxin overexpressing mice. Improved survival was associated with increased expression of the antioxidant enzymes *Gpx1* and *Sod1* as well as the growth factors *Cntf* and *Lif*. Additionally, microglial activation was decreased in these mice. Therefore, support of Müller cell function constitutes a feasible approach to reduce neuronal degeneration after ischemia.

## Introduction

One key element of the pathologic alteration in retinal ischemia/reperfusion injury is the generation of excessive reactive oxygen species (ROS) during reperfusion. Retinal glia (i.e., Müller cells and astrocytes) play a fundamental role in maintaining redox homeostasis and are equipped with tools to resolve redox imbalance^1,2^. Müller cells (MG) are the predominant glia in the vertebrate retina, expand the entire retina forming a structural scaffolding and have cell contacts to all neuronal cell types in the different retinal layers. Therefore they can form a functional unit with a more critical role than for astrocytes, which are restricted to the nerve fiber layer^1^. After injury, Müller cells become reactive and participate in glial scar formation. Interestingly, metabolic support of neurons by Müller cells has been shown to be compatible, even with proliferative reactive gliosis induced in mice^3^. However, morphological and functional alterations in the MG have been found under ischemic conditions or elevated levels of oxidative stress^4^, which probably compromise their supportive role.

Mitochondria are the main source of free radical production in the cell, under both, normal and ischemic conditions^5,6^. Following ischemic injury, endogenous mechanisms of cellular defense are activated to minimize damage. These include superoxide dismutases (SODs), glutathione, vitamins A, C, and E, catalase and other peroxidases. Moreover, recent evidence supports a role for the mitochondrial protein frataxin (FXN) in various diseases, which are caused by increased oxidative stress. FXN deficiency is closely associated with increased oxidative stress damage in Friedreich ataxia (FRDA), an autosomal recessive disease characterized by progressive neurodegeneration in the spinal cord and hypertrophic cardiomyopathy^7–9^. FXN acts as an important regulator of mitochondrial energy metabolism^10,11^ and is involved in several important functions including cellular iron homeostasis and redox balance during oxidative stress^7^. The mature FXN protein is highly expressed in mitochondria of metabolically active tissues such as liver, skeletal and cardiac muscle, and brain^12^. In the adult mouse retina, immunoreactivity to FXN has been shown in the outer and inner plexiform layers, the ganglion cell layer and the inner nuclear layer. Highest levels of FXN were observed in inner segments of the photoreceptor cells^13^.

Improving essential functions of glia was shown to augment glial support to retinal neurons^1^. In particular, support of the glial management of retinal redox homeostasis has been proposed as a therapeutic alternative to overcome neuronal degeneration due to excessive oxidative stress^1^. Because Müller cells span the entire retina and quickly respond to injury, they are ideal targets for therapeutic strategies, including gene therapy^14^. Delivery of exogenous antioxidants or the manipulation of antioxidant pathways within the retina favors the interaction between retinal glia and neurons, and improves RGC survival and function^1,15^. In particular, FXN overexpression was found to stimulate the production of ATP and to induce the activation of antioxidant mechanisms^8^. In a previous study, we found an increased expression of endogenous FXN in the mouse retina 24 hours after an ischemic lesion, indicating that FXN is involved in neuroprotective mechanisms in response to ischemic retinal injury^16^. Furthermore, in the same study, we showed that ubiquitous overexpression of human FXN under the transcriptional control of a human cytomegalovirus (CMV) minimal promoter resulted in neuronal protection. Neuroprotection was associated with changes in expression levels of some stress response molecules, including *Hmox1* and *Hif-2α*, which suggested the participation of glial cells, particularly Müller cells, in FXN-mediated neuroprotection. To further evaluate the cellular mechanisms involved in the FXN-mediated neuroprotection in the ischemic retina and the putative role of Müller cells, here we used a conditional transgenic mouse model overexpressing the human FXN gene specifically in these cells.

## Results

### FXN expression was increased in the retina of naïve MGCre-FXN mice and specifically localized to Müller cells

To evaluate the effect of FXN overexpression in Müller cells on neuronal survival after ischemia, we generated transgenic mice specifically expressing the human FXN cDNA and EGFP transcript in Müller cells (referred to as MGCre-FXN mice, see materials and methods).

**Figure 1:**
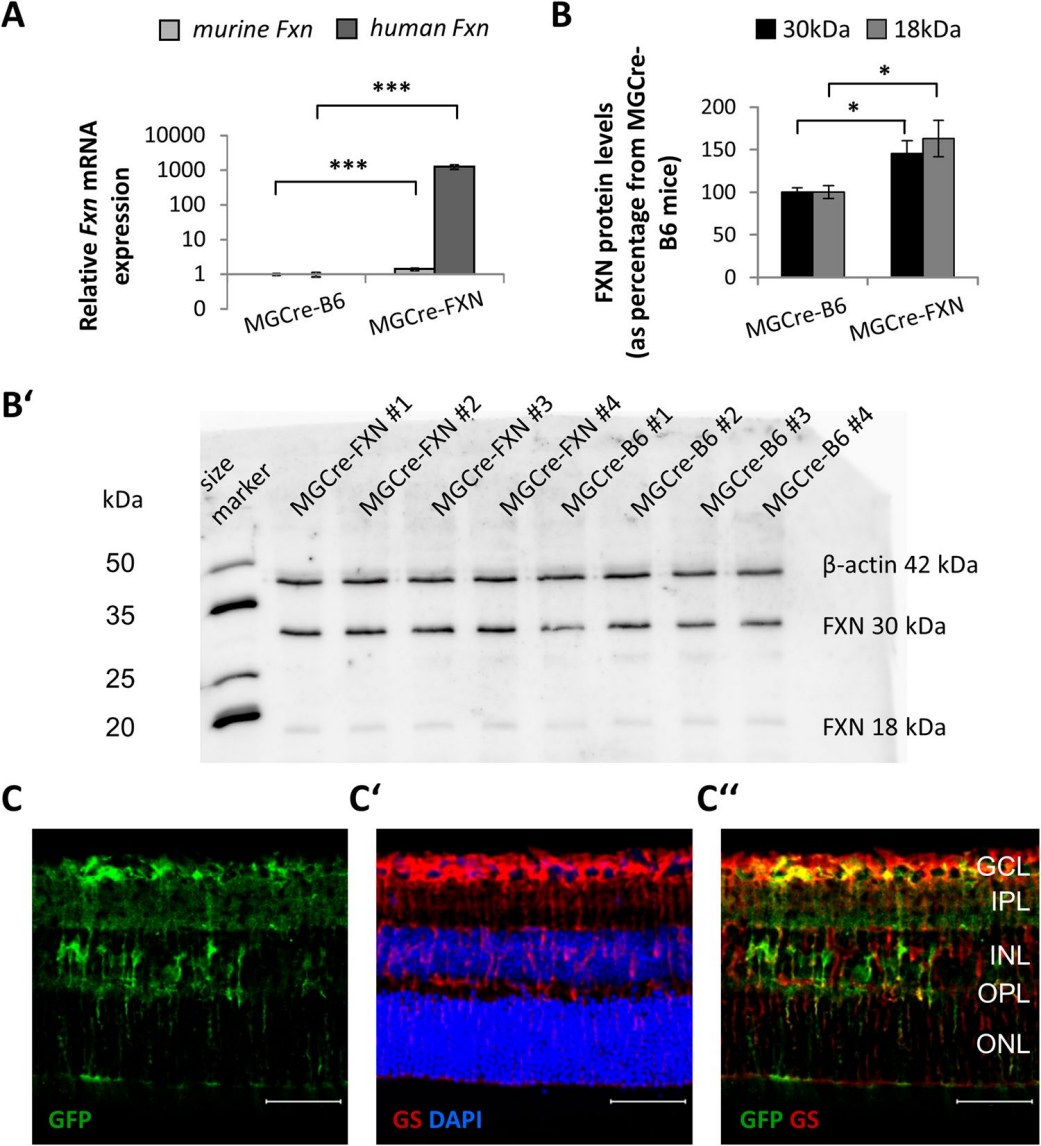
Expression of the human FXN transcript in the retina from MGCre-FXN mice. (**A**) Relative expression of human FXN (*hFXN*) mRNA was determined by quantitative polymerase chain reaction, normalized against *Gapdh* and *Hprt* mRNA levels and depicted as fold increase of expression in MGCre-B6 mice. The y-axis is depicted in logarithmic scaling. Bars represent the mean ± SEM; n = 6; ***P < 0.001. (**B**) FXN protein levels of precursor FXN form (30 kDa) and mature FXN form (18 kDa) were determined by densitometric analysis of Western blotting in MGCre-B6 and MGCre-FXN mice. Values are expressed as a percentage of MGCre-B6 mice and normalized against β-actin. Bars represent the mean ± SEM; n = 4; *P < 0.05. (**B’**) Representative Western blot showing FXN precursor and mature bands for FXN in MGCre-B6 and MGCre-FXN mice. Full-length blot is presented in Supplementary Figure 1. (**C-C”**) GFP expression in MGCre-FXN mice. Immunofluorescent staining of GFP (green), which is co-expressed with hFXN protein, and the Müller cell-specific marker glutamine synthetase (GS, red), as well as DAPI nuclear staining (blue) of retinal sections, were used to illustrate the hFXN location in Müller cells. GCL: ganglion cell layer, IPL: inner plexiform layer, INL: inner nuclear layer, OPL: outer plexiform layer, ONL: outer nuclear layer. Scale bar = 50 μm.

Quantification of mRNA for both, murine and human FXN, showed specific expression of the human FXN transcript in the retina of FXN overexpressing mice (Fig. 1A) and increased levels for endogenous mouse FXN compared with MGCre-B6 mice (1.00 ± 0.05 *vs*. 1.50 ± 0.11-fold increase, *P* < 0.001). The Western blot analysis showed higher levels of expression for both, precursor (30 kDa) and mature FXN protein (18 kDa) in MGCre-FXN mice, compared to MGCre-B6 mice (45% and 65% increase respectively, P < 0.05, Fig. 1B). However, human FXN protein was indistinguishable from the endogenous mouse variant (Fig. 1B’).

To prove the cellular localization of human FXN, retinal slices were co-stained with anti-GFP and the Müller cell-specific marker glutamine synthetase (GS). EGFP was specifically localized in cells spanning the whole retina from FXN overexpressing mice (Fig. 1C). The expression of EGFP was co-localized with the specific Müller cells marker GS in the retina from the MGCre-FXN mice (Fig. 1C–C”) and was detected in 41 ± 8% of all retinal Müller cells.

### Gene expression of oxidative stress-related markers was altered in naïve MGCre-FXN mice

First, we evaluated whether FXN overexpression leads to changes in gene expression in naïve MGCre-FXN mice. In the study we focused on markers well known to be involved in the retinal response and neuronal protection after lesion. These include neurotrophic factors such as leukemia inhibitory factor (*Lif*), nerve growth factor (*Ngf*), brain-derived neurotrophic factor (*Bdnf*), ciliary neurotrophic factor (*Cntf*) and glial cell line-derived neurotrophic factor (*Gdnf*), antioxidant enzymes including glutathione peroxidase 1 (*Gpx1*), superoxide dismutase [Cu-Zn] (*Sod1*), superoxide dismutase [Mn] (*Sod2*), catalase (*Cat*) and heme oxygenase 1 (*Hmox-1*), gliosis and inflammation markers including vimentin (*Vim*), glial fibrillary acidic protein (*Gfap*), glutamate aspartate transporter (*Glast*), glutamine synthetase (*Gs*), tumor necrosis factor alpha (*Tnf-α*) and Interleukin 1 beta (*Il-1β*). We measured the relative mRNA expression by means of qRT-PCR and compared the values against those obtained in naïve non-transgenic MGCre-B6 mice.

**Figure 2:**
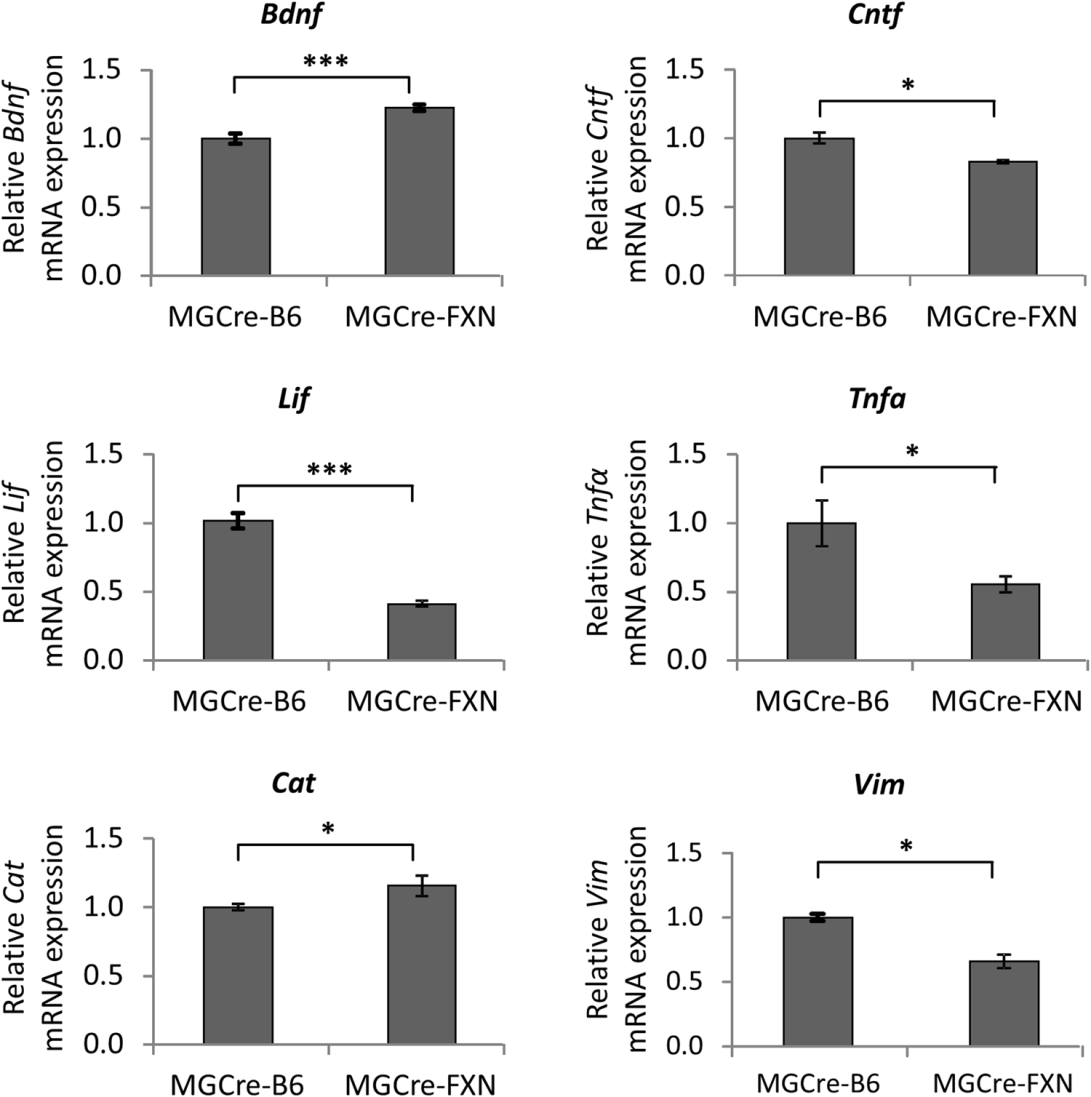
Effect of FXN overexpression on mRNA levels of the neurotrophic factors *Lif*, *Cntf* and *Bdnf*, tumor necrosis factor alpha (*Tnfα*), catalase (*Cat*) and vimentin (*Vim*) in the naïve mouse retina. Bars represent the mean ± SEM, *dashed lines* represent 1.0 ratio, n = 5, *P < 0.05, ***P < 0.001, two-way ANOVA.

The expression levels of the growth factor *Bdnf* were significantly increased, whereas mRNA levels of *Cntf* and *Lif* were decreased in naïve MGCre-FXN mice compared with control MGCre-B6 mice (*Bdnf*: 1.22 ± 0.02-fold increase, *P* < 0.001; *Cntf*: 0.83 ± 0.01-fold decrease, *P* < 0.001; *Lif*: 0.41 ± 0.02-fold decrease, *P* < 0.05, Fig. 2). The inflammation marker *Tnf-α* was significantly decreased in naïve transgenic retinae (0.55 ± 0.06-fold decrease, *P* < 0.05, Fig. 2) and the antioxidative enzyme C*at* was significantly increased (1.16 ± 0.07-fold increase, *P* < 0.05, Fig. 2). Furthermore, the glia marker *Vim*, which is upregulated during gliosis, was decreased in naïve MGCre-FXN mice (0.66 ± 0.05 vs. 1.00 ± 0.03-fold decrease, *P* < 0.05, Fig. 2). The other markers tested did not differ in naïve MGCre-FXN compared with MGCre-B6 mice.

### FXN overexpression in Müller cells increased RGC survival after ischemia/reperfusion

**Figure 3:**
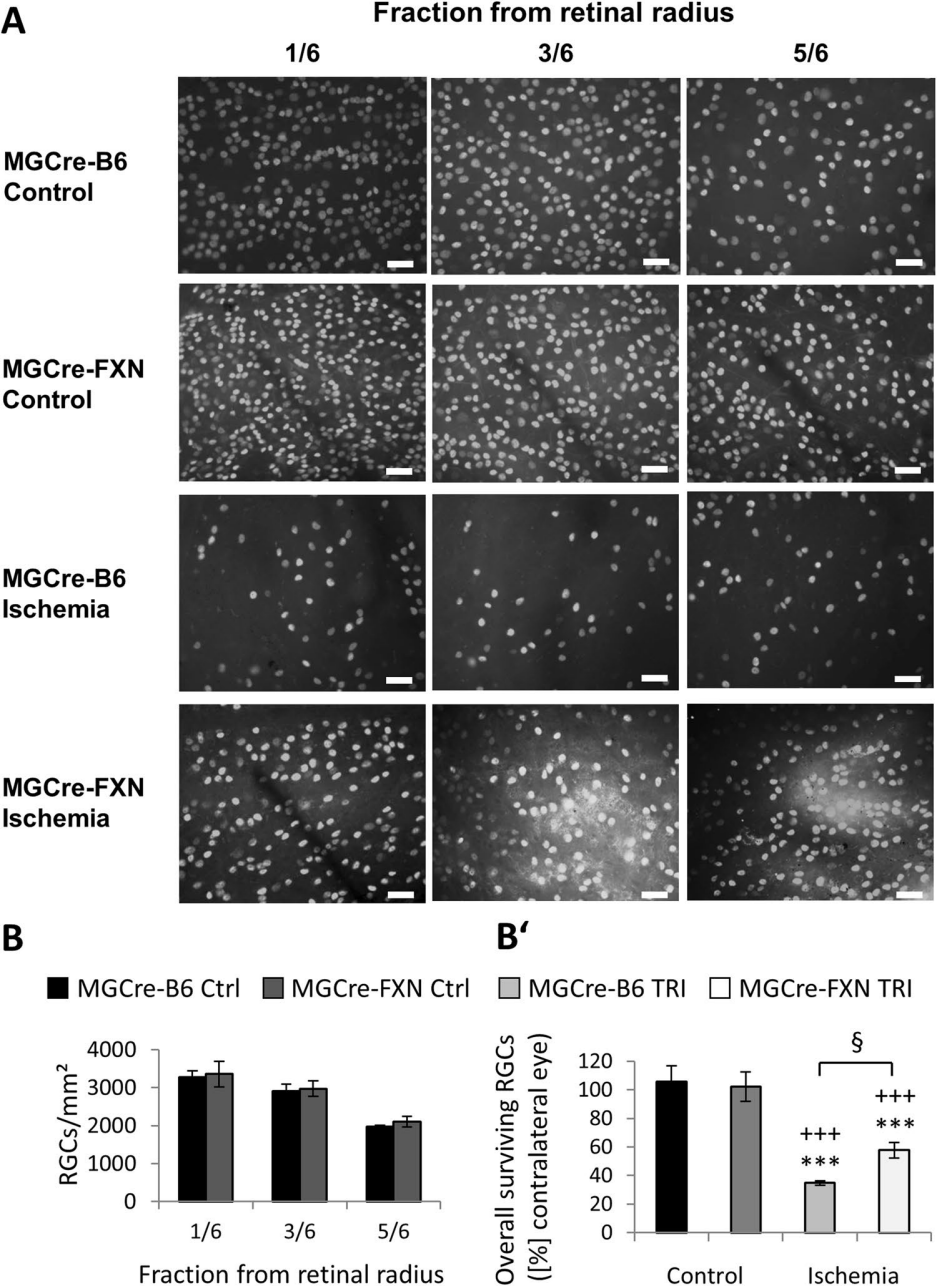
Effect of FXN overexpression in Müller cells on RGC survival 14 days after acute retinal ischemia/reperfusion. (**A**) Images of retinal whole-mount preparations from lesioned and non-lesioned MGCre-B6 and MGCre-FXN animals at various retinal eccentricities (1/6, 3/6, 5/6 from retinal radius) are shown. Images were taken with a fluorescence microscope (x40). Scale bars = 50 µm. (**B**) Analysis of RGCs distribution per retinal eccentricity in non-lesioned MGCre-B6 and MGCre-FXN animals. Numbers of RGCs are expressed as cells/mm², n = 4. (**B’**) Analysis of surviving RGCs after acute retinal ischemia/reperfusion. Numbers of RGCs are expressed as percentages from contralateral eyes. Cell counts were determined 14 days after the insult. Bars represent the mean ± SEM; n = 4–8. ^§^*P* < 0.05, ***P < 0.001 vs. MGCre-B6 Ctrl, ^+++^*P* < 0.001 vs. MGCre-FXN Ctrl.

We next examined whether the expression of FXN in Müller cells increased survival of RGCs after acute ischemia/reperfusion. In naïve retinae from both, MGCre-FXN and MGCre-B6 mice, RGC distribution was highest around the optic nerve (1/6 from retinal radius) and decreased towards the periphery (5/6 from retinal radius). No differences in the overall cell distribution were observed between strains (Fig. 3A,B, Table 1).

**Table 1:** RGC quantification in murine retinal whole mount preparations as a function of the retinal radius.

**Treatment**	**Control**			**retinal ischemia/reperfusion**		
**retinal radius**	**1/6**	**3/6**	**5/6**	**1/6**	**3/6**	**5/6**
**mice strain**		**MGCre-B6**				
**RGC survival**						
cells/mm²	3275 ± 166	2912 ± 180	1975 ± 41	904 ± 99	891 ± 36	1063 ± 58
Percentage vs. contralateral eye	104 ± 11%	98 ± 13%	116 ± 9%	25 ± 3%	29 ± 1%	63 ± 5%
Mean ± SEM	**106 ± 11%**			**35 ± 2%**		
n=		4			6	
**mice strain**		**MGCre-FXN**				
**RGC survival**						
cells/mm²	3258 ± 339	2974 ± 207	2103 ± 139	1358 ± 109*	1416 ± 159*	1098 ± 192
Percentage vs. contralateral eye	96 ± 10%	104 ± 11%	105 ± 12%	54 ± 9%*	54 ± 11%*	60 ± 9%
Mean ± SEM	**102 ± 10%**			58 ± 6% *		
n=	4			8		

After lesion, the number of RGCs decreased in MGCre-B6 and MGCre-FXN mice to 35 ± 2% (*P* < 0.001; n = 6) and 58 ± 6% (*P* < 0.001; n = 8) respectively, compared with non-lesioned control eyes (Fig. 3B’). This represents an increased RGC survival in mice overexpressing FXN of 65% 14 days after ischemia compared with MGCre-B6 mice (*P* < 0.05, Fig. 3B’).

**Figure 4:**
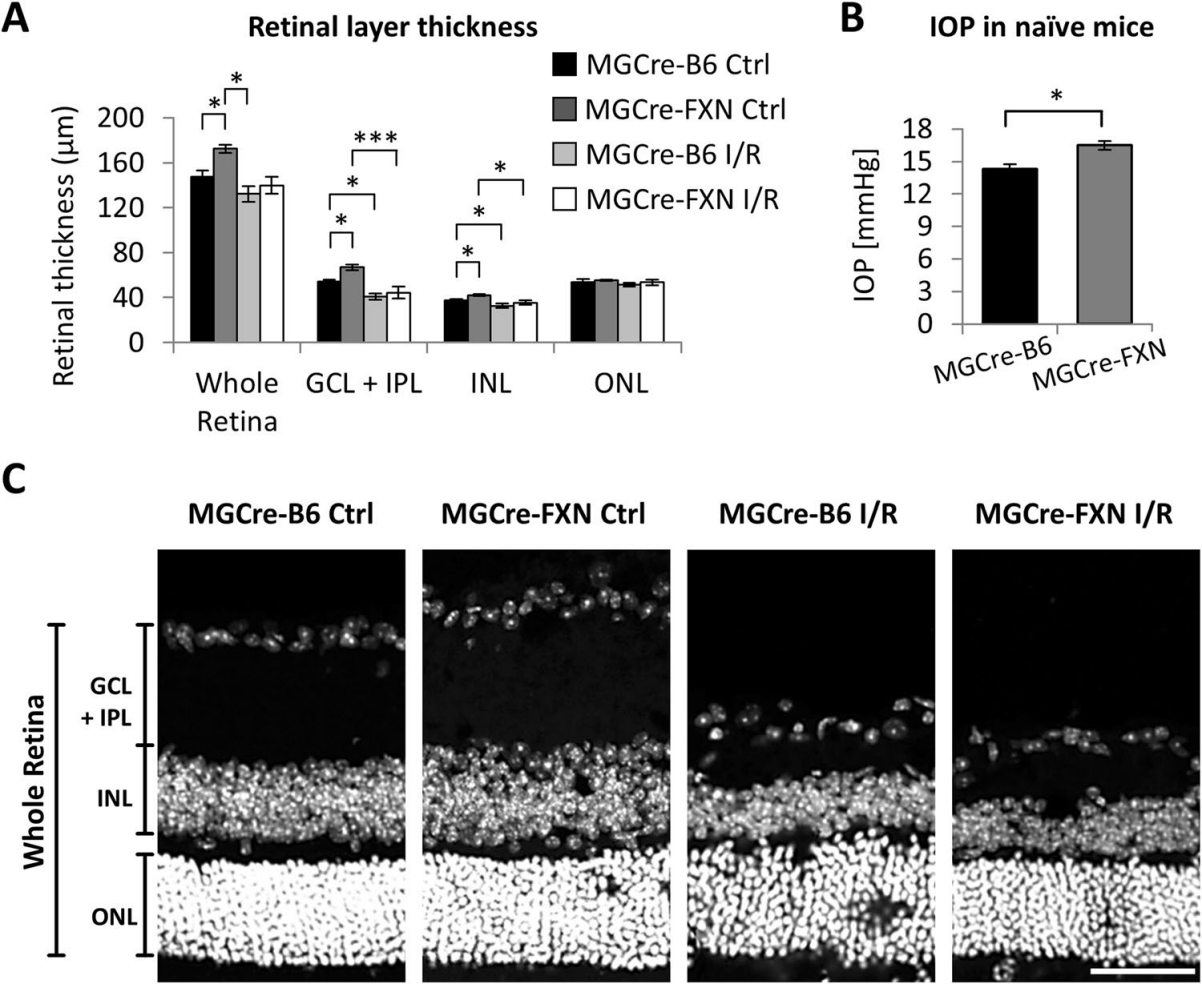
Effects of FXN overexpression on IOP and retinal morphology before and after acute ischemia/reperfusion. (**A**) Analysis of retinal layer thickness in naïve mice and after acute retinal ischemia/reperfusion. Ctrl: control, I/R: ischemia/reperfusion. Bars represent the mean ± SEM; n = 5. *P < 0.05, ***P < 0.001. (**B**) IOP measurement before increased IOP. Bars represent the mean ± SEM; MGCre-B6 n = 16, MGCre-FXN n = 50; *P < 0.05, Mann-Whitney U Rank Sum Test. (**C**) Representative retinal sections through both control and ischemic retinae from MGCre-B6 and MGCre-FXN mice are illustrated. Seven days after ischemia/reperfusion, retinal layers, particularly the IPL, are severely shrunk. GCL: ganglion cell layer, IPL: inner plexiform layer, INL: inner nuclear layer, OPL: outer plexiform layer, ONL: outer nuclear layer. Scale bar = 50 μm.

Interestingly, as illustrated in Fig. 4A, the retinal thickness was increased in non-lesioned MGCre-FXN mice compared with control animals. In particular, thickness of the GCL/IPL, as well as the INL, was increased by 24% and 13%, respectively. This was accompanied by a slight, but significant increase of basal IOP in naïve MGCre-FXN mice (16 ± 0.3 mmHg vs. 14 ± 0.3 mmHg, *P* < 0.05, Fig. 4B).

Retinal ischemia/reperfusion significantly reduced the thickness of the GCL/IPL by approximately 20% on average in MGCre-FXN mice compared with MGCre-B6 animals. Despite an increased neuronal survival, no differences in retinal thickness were found between strains after injury (Fig. 4A/C).

### Increased expression of antioxidant enzymes in MGCre-FXN mice after ischemia/reperfusion

Gene expression ratios of the antioxidant enzymes *Gpx1, Sod1* and *Sod2*, *Cat* and *Hmox1* were evaluated 24 hours after ischemia by means of qRT-PCR to determine whether increased antioxidative capacity might be involved in FXN-mediated neuroprotection.

The expression levels of all enzymes evaluated were significantly increased in MGCre-B6 mice after lesion compared to basal levels (*Gpx1*: 1.60 ± 0.05-fold increase, *P* < 0.001; *Sod1*: 1.16 ± 0.03-fold increase, *P* < 0.001; *Sod2*: 1.20 ± 0.02-fold increase, *P* < 0.001; *Cat*: 1.31 ± 0.02-fold increase, *P* < 0.001; *Hmox1*: 9.44 ± 0.72-fold increase, *P* < 0.001).

**Figure 5:**
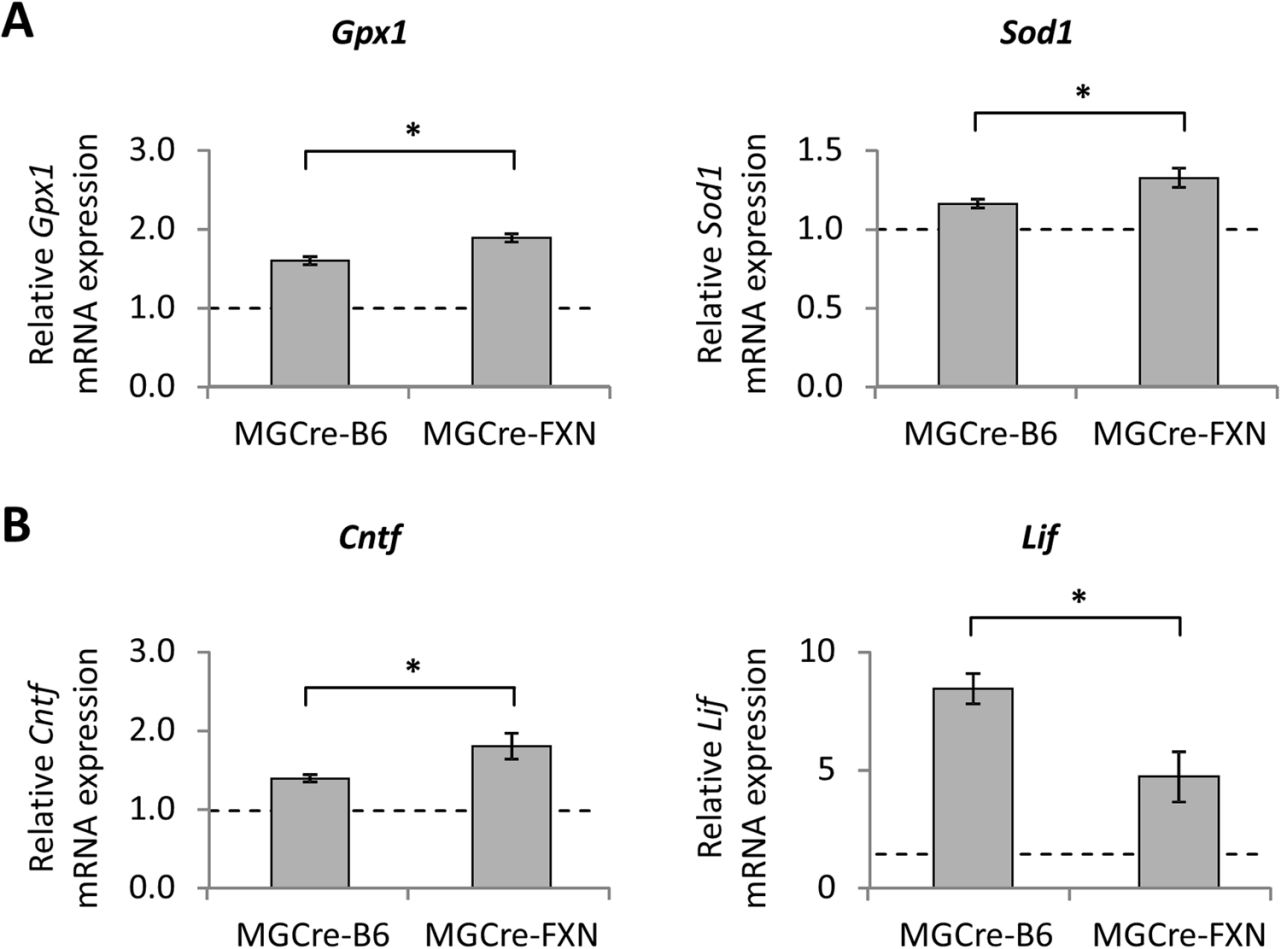
Effect of acute retinal ischemia/reperfusion and FXN overexpression on mRNA levels of (**A**) antioxidant enzymes *Gpx1* and *Sod1* and (**B**) neurotrophic factors *Cntf* and *Lif*. Expression levels of corresponding mRNAs were determined 24 hours after ischemia by quantitative polymerase chain reaction and normalized against *Gapdh* and *Hprt*. Bars represent the mean ± SEM, *dashed lines* represent 1.0 ratio, n = 5, *P < 0.05, two-way ANOVA.

All tested markers also increased above basal levels in MGCre-FXN mice after lesion, (*Gpx1*: 1.89 ± 0.05-fold increase, *P* < 0.001; *Sod1*: 1.33 ± 0.06-fold increase, *P* < 0.001; *Sod2*: 1.16 ± 0.05-fold increase, *P* < 0.001; *Cat*: 1.33 ± 0.03-fold increase, *P* < 0.001; *Hmox1*: 9.44 ± 1.56-fold increase, *P* < 0.001). In addition, the expression levels of *Gpx1* and *Sod1* were significantly higher in MGCre-FXN mice compared with the MGCre-B6 animals after injury (*P* < 0.05; Fig. 5A).

### Expression of neurotrophic factors is altered in MGCre-FXN mice after lesion

To evaluate the role of growth factors in FXN-mediated neuroprotection, changes in expression of the growth factors *Gdnf*, *Ngf*, *Bdnf*, *Cntf* and *Lif* were analyzed 24 hours after ischemia by means of qRT-PCR.

The expression levels of *Ngf*, *Bdnf*, *Cntf* and *Lif* were significantly increased in MGCre-B6 mice (*Ngf*: 1.60 ± 0.11-fold increase, *P* < 0.001; *Bdnf*: 1.43 ± 0.03-fold increase, *P* < 0.001; *Cntf*: 1.39 ± 0.09-fold increase, *P* < 0.001; *Lif*: 8.44 ± 0.65-fold increase, *P* < 0.001).

In MGCre-FXN mice, all measured factors were also increased above basal levels 24 hours after ischemia (*Ngf*: 1.59 ± 0.16-fold increase, *P* < 0.001; *Bdnf*: 1.36 ± 0.03-fold increase, *P* < 0.001; *Cntf*: 1.8 ± 0.16-fold increase, *P* < 0.001; *Lif*: 4.71 ± 1.06-fold increase, *P* < 0.001).

Moreover, *Cntf* expression was higher and *Lif* expression was lower in MGCre-FXN mice compared with MGCre-B6 animals (*P* < 0.05; Fig. 5B).

### Expression levels of inflammation and gliosis marker are not different between MGCre-FXN and MGCre-B6 mice after lesion

Expression of the cytokines *Tnf-α* and *Il-1β* and of the gliosis marker *Glast, Vim, Gs* and *Gfap* were evaluated 24 hours after ischemia by means of qRT-PCR to determine whether inflammation or an altered gliotic response is involved in FXN-mediated neuroprotection.

Pro-inflammatory cytokines were significantly increased in MGCre-B6 mice after lesion compared to basal levels (*Tnf-α*: 6.20 ± 0.42-fold increase, *P* < 0.001; *Il-1β*: 1.73 ± 0.21-fold increase, *P* < 0.001). The expression levels of the intermediary filaments *Gfap* and *Vim* were increased after lesion, as well as the mRNA levels of *Glast* (*Gfap*: 14.04 ± 0.97-fold increase, *P* < 0.001; *Vim*: 5.47 ± 0.31-fold increase, *P* < 0.001; *Glast*: 1.50 ± 0.08-fold increase, *P* < 0.001). *Gs* levels decreased after ischemia (0.28 ± 0.01-fold decrease, *P* < 0.001).

In MGCre-FXN mice, inflammatory markers were also increased above basal levels after lesion (*Tnf-α*: 6,82 ± 0.97-fold increase, *P* < 0.001; *Il-1β*: 1.62 ± 0.48-fold increase, *P* < 0.001). Expression of the intermediary filaments *Gfap* and *Vim* as well as the levels of *Glast* were increased after injury (*Gfap*: 18.03 ± 2.97-fold increase, *P* < 0.001; *Vim*: 4.63 ± 0.53-fold increase, *P* < 0.001; *Glast*: 1.49 ± 0.12-fold increase, *P* < 0.001). *Gs* levels decreased after ischemia (0.30 ± 0.04-fold decrease, *P* < 0.001).

After ischemia, no differences in the expression of these markers were detected between MGCre-B6 background and MGCre-FXN transgenic mice.

### Microglia response was impaired in MGCre-FXN mice after ischemia/reperfusion

Müller cells and microglia communicate via bidirectional signaling that can mediate adaptive responses within the retina following injury^17^. To determine whether FXN expression in Müller cells might influence the microglia response, we evaluated the distribution of Iba1+ microglia and expression of the activation markers CD68 and MHCII in retinal slices before and after lesion. CD68 is a lysosomal protein and can be used to stain microglia in the retina. High expression levels are associated with reactive microglia, while low levels of expression are associated with quiescent ramified microglia^18–20^. In addition, reactive microglia express the major histocompatibility complex class II (MHCII) for antigen presentation^21,22^.

**Figure 6:**
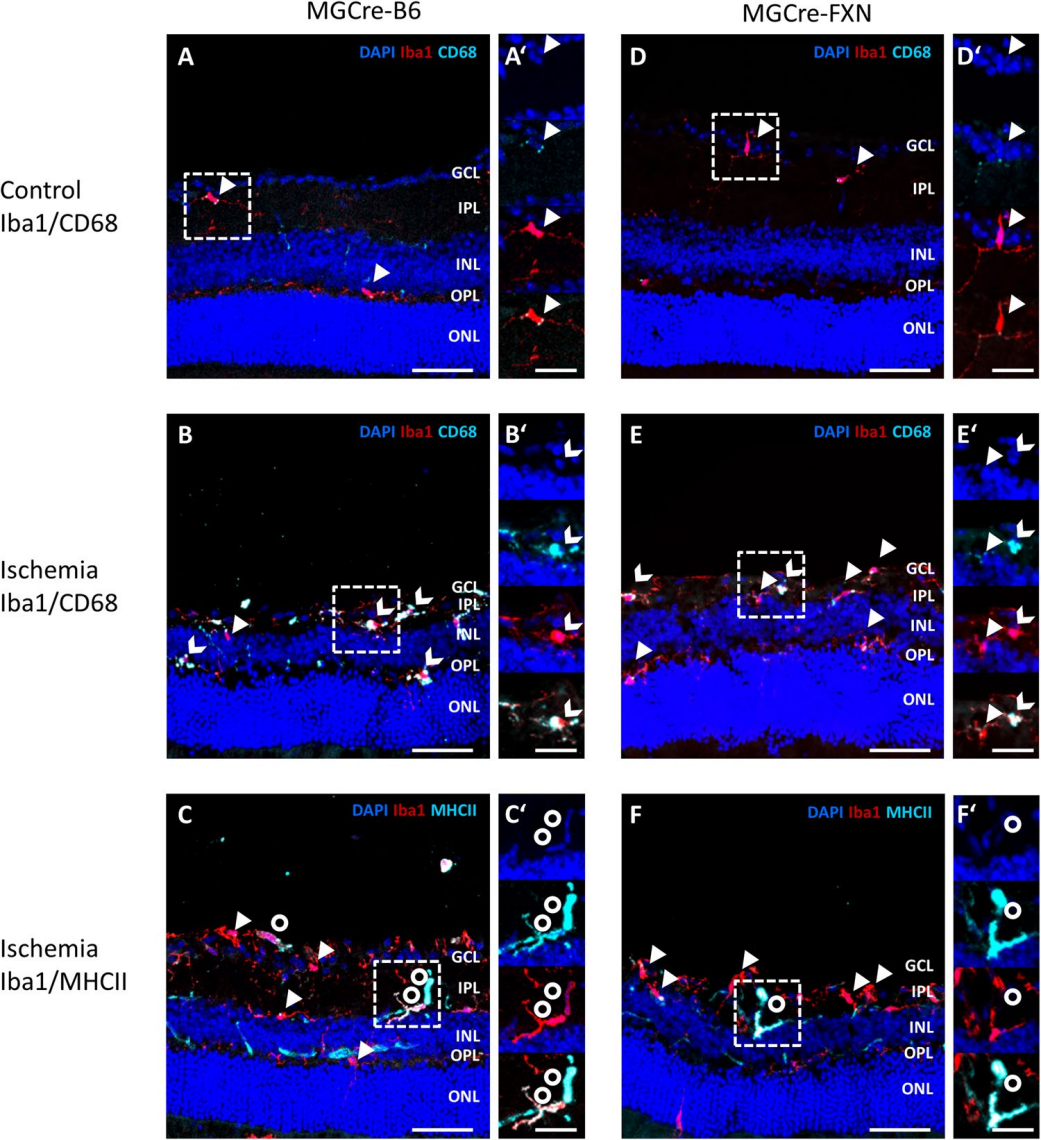
Distribution and localization of reactive microglia in the retina from MGCre-B6 and MGCre-FXN mice. Immunofluorescent staining for Iba1 (red), CD68 (cyan) or MHCII (cyan) as well as DAPI nuclear staining (blue) in retinal sections. Example sections of MGCre-B6 (**A–C**) and MGCre-FXN mice retinae (**D–F**) before and after lesion are shown. Iba1+ microglia (arrowheads) and Iba1+ co-localized with CD68 (notched arrows) or MHCII (circles) were observed. GCL: ganglion cell layer, IPL: inner plexiform layer, INL: inner nuclear layer, OPL: outer plexiform layer, ONL: outer nuclear layer. Scale bar = 50 μm. (**A’–C’**) and (**D’–F’**) show enlarged squares containing example microglia with different merged channels for MGCre-B6 and MGCre-FXN mice. From top to bottom: DAPI; DAPI+ CD68 or MHCII; DAPI+ Iba1; Iba1+ CD68 or MHCII. Scale bar = 25 μm.

**Figure 7:**
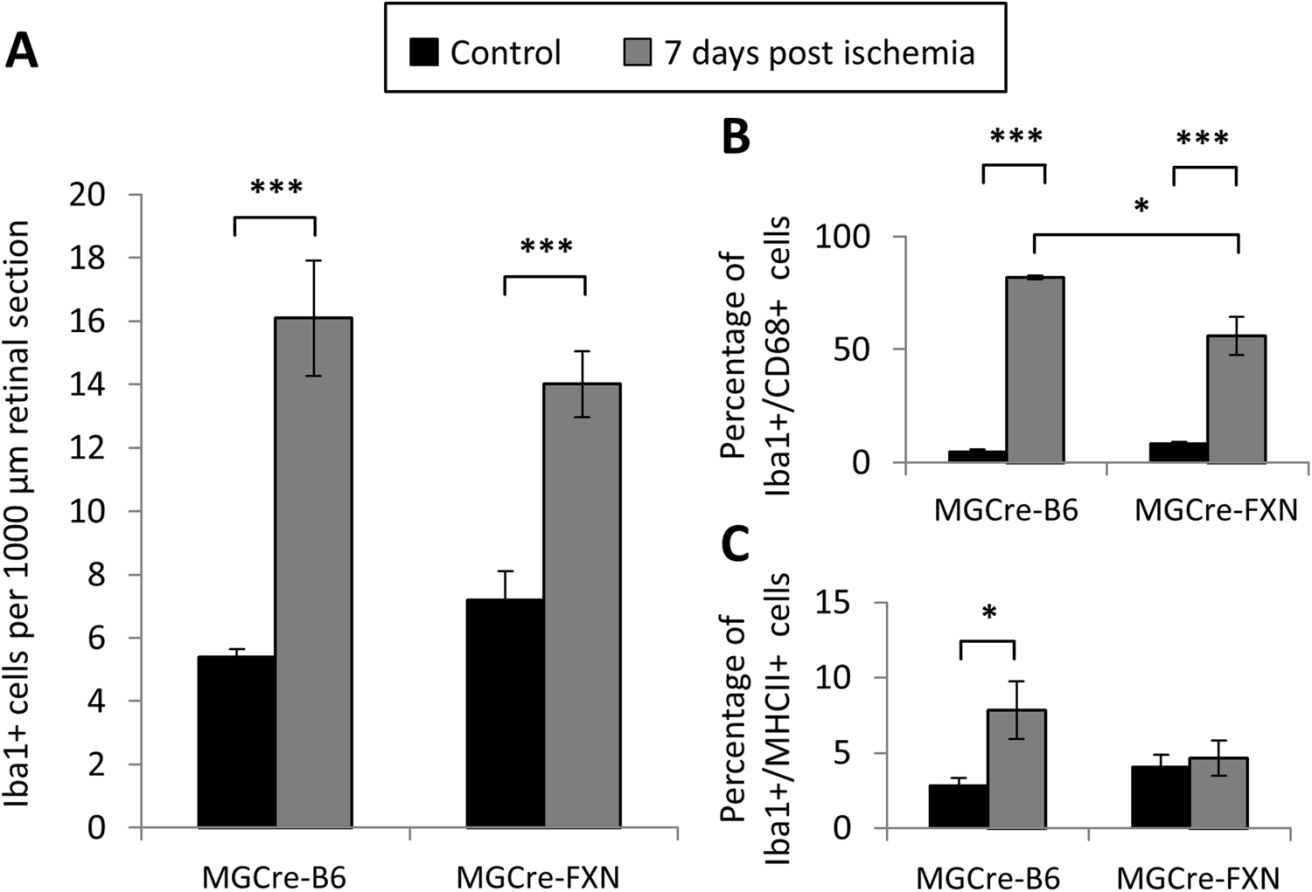
Effect of FXN on the expression of microglial reactivity markers. (**A**) Iba1+ cell counts per retinal section (1000 µm in length) in naïve and lesioned MGCre-B6 and MGCre-FXN mice. Bars represent the mean ± SEM, n = 5, ***P < 0.001, two-way ANOVA. (**B**) Percentage of CD68 positive Iba1 cells in naïve and lesioned MGCre-B6 and MGCre-FXN mice. Bars represent the mean ± SEM, n = 5, *P < 0.05, ***P < 0.001, two-way ANOVA. (**C**) Percentage of MHCII positive Iba1 cells in naïve and lesioned MGCre-B6 and MGCre-FXN mice. Bars represent the mean ± SEM, n = 5, *P < 0.05, two-way ANOVA.

No differences in the number of Iba1+ microglia were observed in naïve retinae of MGCre-B6 and MGCre-FXN mice (16 ± 2 and 14 ± 1 cells/1000 µm, respectively, P < 0.001, Figs 6 and 7A). Few CD68 (4.5 ± 1% and 8.3 ± 1%, respectively) positive and almost no MHCII positive microglia were found (2.8 ± 1% and 4.1 ± 1%, respectively, Figs 6 and 7).

After ischemia, the percentage of Iba1+/Cd68+ microglia strongly increased in both, MGCre-B6 and MGCre-FXN mice; however, in MGCre-FXN mice the number of CD68+ microglia was lower compared with MGCre-B6 mice (56 ± 9% vs. 81.1 ± 1%; P < 0.05; Fig. 7B). The number of MHCII+ microglia after lesion increased only in MGCre-B6 mice (7.8 ± 2% vs. 2.8 ± 1%; P < 0.05; Fig. 7C).

## Discussion

In the present work, we generated a mouse model overexpressing human FXN specifically in retinal Müller cells. Naïve transgenic mice showed slightly decreased expression of gliosis and inflammation markers and of neurotrophic factors. After ischemia, we found an increased RGC survival in transgenic mice overexpressing FXN. Improved cell survival correlated with an increase in gene expression of antioxidant enzymes and neurotrophic factors, whereas the activity of retinal immune cells (microglia) was reduced.

In FXN overexpressing mice the expression levels of *hFXN* mRNA and FXN protein levels were significantly elevated in lysates from the whole retina. These data are similar to results obtained in a previous study using a transgenic mouse model with ubiquitous FXN in all retinal cells^16^. FXN overexpression was specifically increased in about 41% of all Müller cells. This value is similar to data shown in a previous study using the same Cre-recombinase expressing mice model^23^. Naïve transgenic animals did not show differences in the number or distribution of RGCs and microglia as compared with MGCre-B6 animals; however, morphological analysis of the naïve retina from FXN overexpressing mice showed an overall increase in retinal thickness, mainly manifested in the GCL and IPL but also in the INL. We also found a slight but significant increase in basal IOP levels in these animals. Different IOP levels ranging from 11.1 ± 0.5 mmHg to 19.3 ± 0.3 mmHg have been described in several different mouse strains, and have not been related to morphological changes or development of pathologies associated to elevated IOP including glaucoma^24^.

Retinae without Müller cells have decreased resistance to tensile stress rendering the retinal tissue to rip apart, a defect known as retinoschisis^25^. Here, FXN overexpression seems to induce a similar effect by reducing the tensile strength of Müller cells, resulting in expanded retinal layers. The underlying mechanism is unknown, but could involve reduced levels of the intermediate filament vimentin and slight increased IOP levels found in these animals. Vimentin deficiency and mechanical stress have already been associated with a local separation of the inner limiting membrane leading to an increased retinal thickness^26^. Furthermore, it was shown that undisturbed mice deficient for GFAP and vimentin (*GFAP*^*−/−*^ *vim*^*−/−*^) display an altered Müller cell morphology and a decreased resistance to mechanical stress after induced retinal detachment^27^.

There are only a few reports on transgenic mice strains overexpressing FXN and its associated effects. Mice expressing the full-length human FXN cDNA are normal with no signs of ataxia or other obvious abnormalities^11,28^; however, these mice have an altered response during hematopoietic differentiation^28^. The retina was not evaluated in that mouse model. For FXN overexpression, the existing literature presents contradictory results. On the one hand, experiments *in vivo* or *in vitro* in mice revealed that FXN overexpression was innocuous or had a positive effect on cell metabolism, stimulating the production of ATP or activating antioxidant mechanisms^11,16,28–30^. In addition, overexpression of FXN in *Drosophila* promoted cellular resistance to oxidative stress^8^. On the other hand, FXN overexpression can lead to detrimental phenotypes in *Drosophila*, including developmental defects, a decrease in the level of aconitase activity, hypersensitivity to oxidative stress^31^, and reduced viability and impaired embryonic development of muscles and the peripheral nervous system^32^. Interestingly, overexpression of FXN in yeast has also been shown to critically reduce aconitase activity, leading to impaired [Fe-S] cluster assembly and respiration^33^. The mechanisms responsible for these effects are not known, but FXN aggregation and a misfolding of the protein have been shown not to be the cause for the phenotypes that have been observed^32^. Importantly, also in yeast, frataxin overproduction increased cellular antioxidant defenses^33^. In our study, FXN overexpression in Müller cells led to changes in the basal gene expression of several factors. Increased *Cat* might improve retinal homeostasis by providing a less oxidative environment, as also suggested by decreased *Lif* expression^28^. BDNF levels are known to be associated with increased expression of antioxidant proteins and reduced oxidative stress levels^34,35^. Therefore, increased *Bdnf* levels in naïve FXN overexpressing mice might also be indicative of decreased ROS levels, even before lesion onset.

Retinal ischemia/reperfusion injury leads to a high loss of RGCs in MGCre-B6 mice, comparable to values obtained in a previous study from our lab using C57BL/6 J mice^16^. Here, we show for the first time that specific FXN overexpression in Müller cells leads to a significant increase in neuronal survival after lesion. Interestingly, we achieved the same level of neuroprotection compared to transgenic mice with ubiquitous FXN overexpression in the retina^16^. This strongly indicates that Müller cells play a critical role in FXN-mediated neuroprotection after the ischemic lesion.

One of the hallmarks of acute retinal ischemia/reperfusion is the generation of excessive ROS leading to neuronal cell death. Müller cells are well known to protect neuronal cells from oxidative stress by providing antioxidants such as glutathione^36^. Since protective effects mediated by FXN overexpression are known to also involve several antioxidant mechanisms^28,30^, we tested the effect of increased FXN expression on levels of antioxidant enzymes, including *Gpx1*, *Sod1*, *Sod2*, *Cat*, and *Hmox1*. The expression of all enzymes was increased in the retinae of both MGCre-B6 and MGCre-FXN mice after ischemia, in agreement with other studies^2,37,38^. Importantly, *Gpx1* and *Sod1* were higher expressed in transgenic mice than in MGCre-B6 mice. Both factors are already described to be neuroprotective^39,40^. These results are also in agreement with our previous study involving FXN overexpression in the retina, where we found increased levels of *Gpx1*, *Sod1*, *Sod2* and *Cat* transcripts after lesion^16^. This supports our previous findings showing that FXN mediates neuroprotection after ischemia by promoting the antioxidant response. To evaluate further mechanisms involved in neuroprotection by FXN expression in Müller cells, we measured the expression levels of growth factors, including *Bdnf*, *Gdnf*, *Ngf*, *Lif* and *Cntf*. Neurotrophic factors are known to have neuroprotective properties^41^ and have been suggested to be part of an endogenous response mechanism after an ischemic insult^42^. In agreement with other studies, we found an increase in *Bdnf, Ngf*, *Lif* and *Cntf* levels following ischemia-reperfusion in both MGCre-B6 and FXN overexpressing mice^43–47^. Interestingly, in our study, only *Cntf* increased significantly over nontransgenic levels after lesion. CNTF has been described to be neuroprotective for photoreceptor cells^48–50^ and RGCs in several studies^51–54^. In the retina, CNTF is expressed by various cell types and particularly by Müller cells^55^. Increased *Cntf* transcripts strongly support an important role of Müller cells in FXN-mediated neuroprotection. In contrast, *Lif* transcripts did not reach the expression level of nontransgenic animals after lesion. However, it was significantly increased above transgenic basal levels. LIF is an important endogenous neuroprotective factor in the retina^56,57^. Specifically, photoreceptor injuries induce a subset of Müller cells to express Lif^56^. Further, it’s transcript seems to be stabilized by H_2_O_2_^58^. Together with the increased antioxidative response, these results indicate that FXN potentiates Müller cell-mediated neuroprotection after acute retinal ischemia/reperfusion by increasing the neurotrophic support.

Our results show a decreased microglia response after transient ischemia in transgenic mice overexpressing FXN in Müller cells. In models of RGC damage, the extent of microgliosis negatively correlates with the number of surviving RGCs^59^. A decreased number of microglia would be expected with increased neuronal survival; however, we did not observe differences in microglial numbers after lesion in nontransgenic and transgenic mice. Interestingly, the number of reactive microglia, as indicated by the expression of CD68 and MHCII, was decreased in FXN overexpressing mice after ischemia. CD68 and MHCII are markers of microglia activation reflecting phagocytic and immune activity, respectively^19–22,60,61^. Microglia reactivity and inflammation are common hallmarks of a broad spectrum of retinal diseases and contribute to RGC loss associated with ON damage in glaucoma^62^ and retinal ischemia^63^. Blocking microglia reactivity is known to be neuroprotective e.g. in experimental glaucoma^64–66^. Our results are in agreement with studies showing that the expression of MHCII is upregulated in activated microglia during axonal degeneration induced by ocular hypertension in rats^67,68^ and downregulated MHCII is associated with less severe axonal degeneration in a model of glaucoma by chronic increased IOP^68^. Reduced expression levels of MHCII may inhibit the activation of invading T-cell, thereby diminishing the inflammatory cascade leading to tissue damage^69,70^. Decreased CD68 positive microglia might reflect a reduced phagocytic activity associated with an increased neuronal survival in MGCre-FXN mice after lesion. The phagocytic activity of microglia is associated to clearance of cells debris, and of damaged or dying cells^61^. On the other hand, Müller cells and microglia actively cross-talk to maintain constant levels of neurotrophic factors important for retinal physiology^71,72^. Microglia can directly trigger the release of several neurotrophic factors from Müller cells, including GDNF, LIF, CNTF and NGF^17,56,73,74^. Furthermore, vimentin expression serves as a marker for activated states of microglia^18,19,75^. In our study, FXN-overexpressing Müller cells seemed to regulate microglial function under physiological conditions, as suggested by reduced vimentin expression in naïve retinae. We propose that FXN-mediated modulation of the Müller cell response to lesion impacts microglial reactivity, possibly by reducing the number of cells in the reactive M1-state, as seen by unchanged expression of MHCII and decreased number of CD68 positive cells in MGCre-FXN mice following injury^61,76^.

Taken together, our results show that FXN overexpression in Müller cells improves RGC survival after acute ischemia/reperfusion injury and this is associated with an increased expression of antioxidant enzymes and neurotrophic factors. Furthermore, our study highlights Müller cells as important players in retinal pathologies and indicates that support of the glial management of retinal redox homeostasis might constitute a therapeutic alternative to overcome neuronal degeneration due to increased oxidative stress.

## Materials and Methods

### Animal Guidelines

All experiments were performed in accordance with the European Convention for Animal Care and with the ARVO Statement for the Use of Animals in Ophthalmic and Vision Research and were also approved by the local animal care committee (Thüringer Landesamt für Verbraucherschutz (TLV) - Abteilung 2 Gesundheitlicher und technischer Verbraucherschutz, accreditation numbers: 02-047/08 and 02-013/11). Animals were housed in standard cages in groups of 5 animals on a 14-hour light/10-hour dark cycle, with food and water available *ad libitum*, a temperature ranging from 22 °C to 25 °C and humidity ranging from 55% to 60%.

Male C57BL/6 J background and transgenic mice overexpressing FXN, weighing 25 ± 2 g, aged 12 ± 1 weeks, were used in the study. Transgenic animals overexpressing FXN in Müller cells were generated by breeding homozygous mice carrying cassettes of human PVMD2-rtTA and TRE-cre (PVMD2TREcre^23,77,78^, provided by Dr. Yun-Zheng Le, Department of Cell Biology, University of Oklahoma Health Sciences Center, Oklahoma City, USA) to heterozygous mice carrying a CMV-driven human FXN cDNA preceded by a loxP-flanked stop cassette (R26CMVFxn/wt, provided by Prof. Michael Ristow, Department of Health Sciences and Technology, Swiss Federal Institute of Technology, Zürich, Switzerland), hereafter referred to as MGCre-FXN. Cre-mediated excision of the stop cassette leads to expression of the FXN gene and of green fluorescent protein (EGFP), as has been shown in our previous work with ubiquitous FXN overexpressing mice^16^. Background mice were generated by breeding C57BL/6 J with PVMD2TREcre mice, hereafter referred to as MGCre-B6. Mice were randomized for each experimental setup into a non-lesioned group (MGCre-B6 n = 22; MGCre-FXN n = 22) or an ischemia group (MGCre-B6 n = 16; MGCre-FXN n = 18).

### Retinal ischemia/reperfusion injury

Retinal ischemia/reperfusion injury was performed as previously described^16,79^. Briefly, mice were anesthetized with an intraperitoneal injection of 5% chloral hydrate in phosphate buffered saline (PBS; 500 mg/kg body weight; Fluka, Seelze, Germany). After application of topical anesthesia (4 mg/ml oxybuprocaine-hydrochloride; Bausch & Lomb GmbH, Feldkirchen, Germany), the anterior chamber of the right eye was cannulated with a 30-gauge needle connected to an elevated normal saline reservoir. Intraocular pressure (IOP) was elevated above systolic pressure (increased from 15.5 ± 2.6 mmHg to 93 ± 4.4 mmHg) for 45 minutes. IOP was measured using an induction/impact tonometer (TonoLab; Tiolat Ltd., Helsinki, Finland). This method has been validated for the mouse eye^80^. One drop of antibiotic solution (ofloxacin; Bausch & Lomb GmbH, Berlin, Germany) was applied topically to the treated eye after cannulation. After 45 minutes of ischemia, the needle was withdrawn and the IOP normalized. Treated eyes were inspected daily and animals with signs of inflammation or iatrogenic cataract were excluded from the study. Animals for RGC evaluation were sacrificed with an overdose of chloral hydrate (30%) 14 days after ischemia, as previously described^16^. For microglia analysis, mice were sacrificed 7 days after lesion, coinciding with highest microglial proliferation as already reported^59^. For qRT-PCR analysis, mice were sacrificed 24 hours post ischemia as described in a previous study^16^.

### Retinal tissue fixation and sectioning

To localize human FXN (hFXN) expression and evaluate microglia response after ischemic injury, retinal sections from background and MGCre-FXN mice were prepared. Briefly, animals were sacrificed with an overdose of chloral hydrate (30%), eyes were removed and fixed in 4% PFA for 20 min at room temperature (RT), enucleated, again fixed in 4% PFA for 20 min, washed in PBS and cryoprotected by immersion in 30% sucrose in PBS overnight at 4 °C. Eyecups were then frozen in an appropriate embedding medium (Tissue Tek; Sakura, Loeterwoude, The Netherlands) and cryosectioned into 16 µm slices.

Retinal whole mounts of MGCre-B6 and MGCre-FXN mice were prepared to evaluate RGC survival following ischemia. Eyes were enucleated and retinae were removed and fixed by immersion in 4% PFA for 20 min. After washing in PBS, retinae were flattened by making incisions from the periphery halfway to the optic nerve to form four symmetric lobes.

### Immunofluorescent staining

Immunofluorescent staining protocols were similar for retinal slices and whole mounts. Retinal sections were dried at 37 °C for 45 min and fixed with 4% PFA for 20 min. Retinal whole mounts were permeabilized with 0.3% Triton X-100 in PBS for 45 min at RT. Blocking was achieved by incubation with 3% bovine serum albumin (BSA) and 10% normal donkey serum (NDS) in PBS supplemented with 0.3% Triton X-100 for 2 hours at RT. After non-specific binding was blocked, whole mounts were incubated with primary antibody raised against the RGC-specific transcription factor Brn3a (goat anti-Brn3a, 1:300; Santa Cruz, Heidelberg, Germany), whereas slices were incubated with primary antibodies directed against ionized calcium-binding adaptor molecule (rabbit anti-Iba1, 1:500; Wako, Neuss, Germany), cluster of differentiation 68 (rat anti-CD68, 1:200; Serotec, Düsseldorf, Germany), major histocompatibility complex class II (rat anti-MHCII, 1:10,000; affymetrix, CA, USA), GFP (goat anti-GFP, 1:100, Acris, Herford, Germany) and glutamine synthetase (mouse anti-GS, 1:250, Millipore, Darmstadt, Germany) in 5% NDS, overnight at 4 °C. After washing with PBS, probes were incubated with corresponding secondary antibodies (Molecular Probes, Leiden, The Netherlands) in 10% NDS for one hour at RT. For nucleic acid staining, the retinal slices were immersed into a DAPI (4′-6-Diamidino-2-phenylindole) solution for 5 min. Specificity of the staining was tested by incubation without primary antibody.

### Fluorescence microscopy

RGC survival was evaluated on retinal whole mounts 14 days post-ischemia. Brn3a-labeled RGCs were counted in single fields at three different retinal eccentricities in each of the four whole mount lobes (1/6, 3/6 and 5/6 from retinal radius; 0.093 mm^2^ each, 12 fields in total) according to Schmeer *et al*.^81^ and Schultz *et al*.^16^. The number of surviving RGCs was expressed as the number of cells per square millimeter and cell counts were given as the percentage of corresponding contralateral and non-lesioned eyes.

The number of GFP+ Müller cells specifically expressing FXN was determined on slices from MGCre-FXN mice by counting GS+ GFP+ cell processes located in the IPL. The results are given as percentage from the whole GS+ cell population.

The number of Iba1+ microglia was determined on retinal sections. Cell counts were corrected for slice length and expressed as cell count per 1000 µm. The number of reactive microglia was assessed by counting Iba1+ CD68+ and Iba1+MHCII+ cells.

Image acquisition and cell counting was performed by fluorescence microscopy (40x magnification, Axioplan2 Imaging microscope; Carl Zeiss Meditec, Jena, Germany) on retinal whole mounts and by confocal laser scanning microscopy using the ZEN software (40× magnification, 710 Meta; Carl Zeiss Meditec) on retinal slices. Experimenters were blinded for animal genotype and treatment.

### Quantitative polymerase chain reaction analysis

Eyes were enucleated 24 hours post ischemia and retinae were shock frozen in liquid nitrogen. Isolation of mRNA was carried out using standard protocols with the QIAzol lysis reagent (Qiagen, Hilden, Germany). RNA concentration and purity were determined by spectrophotometry (NanoDrop 2000c; Peqlab/VWR, Darmstadt, HE, Germany). DNA contamination and RNA integrity, as assessed by the RNA Integrity Score, were determined using the QIAxcel RNA QC Kit v2.0 (Qiagen) with the QIAxcel Advanced system (Qiagen). Equal amounts of total retinal mRNA (100 ng/μl) were reversely transcribed into cDNA using a Revert Aid First Strand cDNA Synthesis Kit (Thermo Scientific, Schwerte, Germany). Real-time qPCR was carried out with Brilliant II SYBR Green QPCR Mastermix (Agilent Technologies, Santa Clara, CA, USA). A final cDNA concentration of 25 ng per 20 μl of sample volume including 500 nM specific primers was used. After denaturing at 95 °C for 10 min, 40 amplification cycles were carried out as follows: denaturation at 95 °C for 60 s, annealing at 60 °C for 30 s, and elongation at 72 °C for 30 s.

Primer sequences were selected/designed to exclusively recognize specific target sequences using the Primer-BLAST online tool^82^. The primer specificity and predicted target size were confirmed by capillary electrophoresis. The primer sequences for murine (*mFxn*) and human FXN (*hFXN*), antioxidant enzymes, neurotrophic factors and inflammatory cytokines are depicted in Table 2. Glyceraldehyde 3-phosphate dehydrogenase (*Gapdh)* and hypoxanthine-guanine phosphoribosyltransferase (*Hprt)* were used as housekeeping genes. Relative expression ratios (fold changes) of the target genes were calculated using the Pfaffl method^83^.

**Table 2:** Primer sequences for mRNAs analyzed in the study, together with the expected product length.

**Gene name**		**Primer Seq. (5’-** > **3’)**	**Prod. size (bp)**
***hFXN***	Fw	CCTTGCAGACAAGCCATACA	150
human Fratxin	Rev	CCACTGGATGGAGAAGATAG	
***Fxn***	Fw	CCTGGCCGAGTTCTTTGAAG	152
mouse FXN	Rev	GCCAGATTTGCTTGTTTGG	
**Antioxidant response**			
***Gpx1***	Fw	GGGACTACACCGAGATGAACGA	197
glutathione peroxidase 1	Rev	ACCATTCACTTCGCACTTCTCA	
***Sod1***	Fw	GTCCGTCGGCTTCTCGTCT	163
superoxide dismutase [Cu-Zn]	Rev	CACAACTGGTTCACCGCTTG	
***Sod2***	Fw	ATTAACGCGCAGATCATGCA	161
superoxide dismutase [Mn]	Rev	TGTCCCCCACCATTGAACTT	
***Cat***	Fw	GCAGATACCTGTGAACTGTC	229
catalase	Rev	GTAGAATGTCCGCACCTGAG	
***Hmox1***	Fw	GGTGATGGCTTCCTTGTACC	155
heme oxygenase 1	Rev	AGTGAGGCCCATACCAGAAG	
**Glial response**			
***Vim***	Fw	TGAAGGAAGAGATGGCTCGT	194
Vimentin	Rev	GGTGTCAACCAGAGGAAGTGA	
***Gfap***	Fw	AGAAAGGTTGAATCGCTGGA	176
glial fibrillary acidic protein	Rev	GCCACTGCCTCGTATTGAGT	
***Glast***	Fw	GCCCTCCGACCGTATAAAAT	128
Glutamate aspartate transporter	Rev	GCCATTCCTGTGACGAGACT	
***Gs***	Fw	ATCGGGTGTGCGAAGACTTT	181
glutamine synthetase	Rev	CGAATGTGGTACTGGTGCCT	
**Neurotrophic factors**			
***Lif***	Fw	AATGCCACCTGTGCCATACG	216
leukemia inhibitory factor	Rev	CAACTTGGTCTTCTCTGTCCCG	
***Ngf***	Fw	AGCATTCCCTTGACACAG	99
nerve growth factor	Rev	GGTCTACAGTGATGTTGC	
***Bdnf***	Fw	TGGCTGACACTTTTGACCAC	131
brain-derived neurotrophic factor	Rev	CAAAGGCACTTGACTGCTGA	
***Cntf***	Fw	ATGACTGAGGCAGAGCGACT	157
ciliary neurotrophic factor	Rev	AGGCAGAAACTTGGAGCGTA	
***Gdnf***	Fw	TGGGCTATGAAACCAAGGAG	142
glial cell line-derived neurotrophic factor	Rev	CAACATGCCTGGCCTACTTT	
**Inflammatory markers**			
***Tnf-alpha***	Fw	GTCTACTGAACTTCGGGGTGAT	102
Tumor necrosis factor alpha	Rev	ATGATCTGAGTGTGAGGGTCTG	
***Il1b***	Fw	GAAGAGCCCATCCTCTGTGA	96
Interleukin 1 beta	Rev	TTCATCTCGGAGCCTGTAGTG	
**Housekeeping genes**			
***Hprt***	Fw	TGACACTGGTAAAACAATGCA	94
hypoxanthine-guanine phosphoribosyltransferase	Rev	GGTCCTTTTCACCAGCAAGCT	
***Gapdh***	Fw	AGGTCGGTGTGAACGGATTTG	123
Glyceraldehyde 3-phosphate dehydrogenase	Rev	TGTAGACCATGTAGTTGAGGTCA	

fw, forward primer; rev, reverse primer.

### Western blotting

To evaluate the protein levels of retinal FXN in MGCre-B6 and MGCre-FXN mice, Western blotting was performed as previously described^16^. Primary antibodies raised against FXN (1:500; Santa Cruz), *β*-Actin (1:10,000; abcam, Cambridge, UK) and horseradish peroxidase (HRP)-conjugated secondary antibody (1:5000 dilution) were used. Protein bands were visualized using an enhanced chemiluminescence reaction kit (Immun-Star WesternC Chemiluminescence Kit, BioRad, München, Germany), photographed with Fujifilm LAS-3000 Imager and analyzed with ImageJ software (1.46 v). Measurements were done in duplicate. Each band was normalized against the corresponding *β*-actin band. Changes in protein expression were expressed as a percentage of control mice.

### Statistical Analysis

All results are expressed as the mean ± standard error of the mean (SEM). Each group consisted of at least four animals. Proof of normal distribution was done for all data by a Shapiro-Wilk test. For RGC survival analysis, statistical significance was assessed by using one-way ANOVA followed by Holm-Sidak post hoc analysis and comparing MGCre-B6 control mice and treatment groups. For relative gene expression analysis, ratio data were log2 transformed and two-way ANOVA followed by Holm-Sidak post hoc analysis was applied for comparison of lesion effects between mice strains. For microglia cell counts and reactivity marker expression, two-way ANOVA followed by Holm-Sidak post hoc analysis was applied for comparison of lesion effects between mice strains. For Western blot analysis, the results were compared by one-way ANOVA followed by Holm-Sidak post hoc analysis comparing background and transgenic mice. SigmaPlot (13.0 v) was used and the level of significance was set at *P* < 0.05.

### Data availability

The datasets generated during and/or analyzed during the current study are available from the corresponding author on reasonable request.

## Electronic supplementary material

Supplementary Figure 1
