# Supplementary material for: Frataxin overexpression in Müller cells protects retinal ganglion cells in a mouse model of ischemia/reperfusion injury in vivo
Source: Sci Rep. 2018 Mar 19;8:4846. doi: 10.1038/s41598-018-22887-5 (PMC5859167; doi:10.1038/s41598-018-22887-5)

Frataxin overexpression in Müller cells protects retinal ganglion cells in a mouse model of acute glaucoma *in vivo*.

Rowena Schultz[1], Melanie Krug[2], Michel Precht[2], Stefanie G. Wohl[3], Otto W. Witte[2], Christian Schmeer[2,*]

[1]*Department of Ophthalmology, Jena University Hospital, Jena, Germany.*

[2]*Hans-Berger Department of Neurology, Jena University Hospital, Jena, Germany.*

[3]*Department of Biological Structure, University of Washington Seattle, Seattle, United States.*

*Corresponding author at: Hans-Berger Department of Neurology, Jena University Hospital, Jena 07747, Germany. Tel.: 49 36419325828. E-mail: christian.schmeer@med.uni-jena.de

Supplementary information

Supplementary Figure 1: Representative Western blot showing FXN precursor and mature bands for FXN in MGCre-B6 and MGCre-FXN mice, full-length blot.

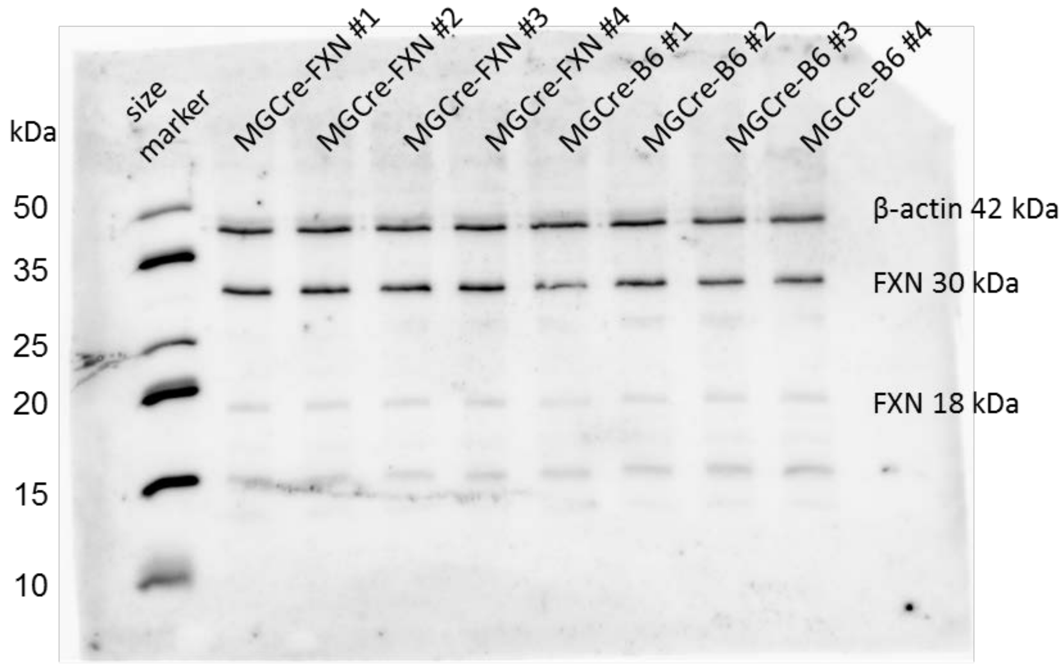

Supplement: Supplementary file 1 — Supplementary Figure 1 [file 41598_2018_22887_MOESM1_ESM.pdf]
